# Development and validation of multivariable quantitative ultrasound for diagnosing hepatic steatosis

**DOI:** 10.1038/s41598-023-42463-w

**Published:** 2023-09-14

**Authors:** Sun Kyung Jeon, Jeong Min Lee, Soo Jin Cho, Young-Hye Byun, Jae Hwan Jee, Mira Kang

**Affiliations:** 1 Department of Radiology, Seoul National University Hospital and Seoul National University College of Medicine, 101 Daehangno, Jongno-gu, Seoul, 03080 Korea; 2 Institute of Radiation Medicine, Seoul National University Medical Research Center, Seoul, Korea; 3 Center for Health Promotion, Samsung Medical Center, Sungkyunkwan University School of Medicine, 81 Irwon-ro, Gangnam-Gu, Seoul, 06351 Korea; 4 Department of Digital Health, Samsung Advanced Institute of Health Sciences & Technology (SAIHST), Sungkyunkwan University, Seoul, Korea; 5 Digital Innovation Center, Samsung Medical Center, Sungkyunkwan University School of Medicine, Seoul, Korea

**Keywords:** Health care, Medical research

## Abstract

This study developed and validated multivariable quantitative ultrasound (QUS) model for diagnosing hepatic steatosis. Retrospective secondary analysis of prospectively collected QUS data was performed. Participants underwent QUS examinations and magnetic resonance imaging proton density fat fraction (MRI-PDFF; reference standard). A multivariable regression model for estimating hepatic fat fraction was determined using two QUS parameters from one tertiary hospital (development set). Correlation between QUS-derived estimated fat fraction(USFF) and MRI-PDFF and diagnostic performance of USFF for hepatic steatosis (MRI-PDFF ≥ 5%) were assessed, and validated in an independent data set from the other health screening center(validation set). Development set included 173 participants with suspected NAFLD with 126 (72.8%) having hepatic steatosis; and validation set included 452 health screening participants with 237 (52.4%) having hepatic steatosis. USFF was correlated with MRI-PDFF (Pearson r = 0.799 and 0.824; development and validation set). The model demonstrated high diagnostic performance, with areas under the receiver operating characteristic curves of 0.943 and 0.924 for development and validation set, respectively. Using cutoff of 6.0% from development set, USFF showed sensitivity, specificity, positive predictive value, and negative predictive value of 87.8%, 78.6%, 81.9%, and 85.4% for diagnosing hepatic steatosis in validation set. In conclusion, multivariable QUS parameters-derived estimated fat fraction showed high diagnostic performance for detecting hepatic steatosis.

## Introduction

The global burden of metabolic dysfunction-associated steatotic liver disease (MASLD) is substantial and has an increasing prevalence worldwide, affecting up to 25% of the general population^1,2^. MASLD is emerging as an important cause of end-stage liver disease and hepatocellular carcinoma^3^. Furthermore, patients with MASLD and concomitant evidence of metabolic dysfunction-associated steatohepatitis (MASH) and advanced fibrosis are at markedly increased risk of adverse outcomes, including overall mortality and liver-specific morbidity and mortality, respectively^4^. Therefore, it is clinically crucial to detect MASLD early when fat accumulation in hepatocytes is potentially reversible by effective lifestyle modification and prevent the progression of MASLD to MASH or liver cirrhosis^5^.

Ideally, the diagnostic tools for MASLD should be able to diagnose and quantify steatosis. Both liver biopsy and magnetic resonance imaging proton density fat fraction (MRI-PDFF) are widely accepted as reference standards for evaluation of hepatic steatosis^2^. However, the invasiveness and sampling errors of biopsy as well as the high cost and limited accessibility of MR scanners limit their use as first-line diagnostic test for screening MASLD^6,7^. On the contrary, ultrasound (US) has better potential as screening test for MASLD than biopsy or MRI-PDFF, as it has several advantages including its affordability, rapidity, and accessibility on a global scale^1^. However, as qualitative assessment of B mode US echogenecity is likely dependent on the manufacturer, transducer, frequency, and experience of the operator, it can be negatively affected by interobserver variability^8,9^.

In recent years, various pulse echo quantitative ultrasound (PE-QUS) biomarkers, including ultrasound attenuation, backscatter coefficient, and speed of sound, have been proposed as objective tools with good reproducibility for hepatic steatosis quantification^8–16^. More recently, a few studies proposed US-derived fat fraction that combines attenuation and backscatter quantification using various mathematical approaches such as multivariable regression model or convolutional neural networks (CNNs)^17–20^, which presented measurement results in percentage, and showed good correlation with MRI-PDFF. This development is crucial because the quantitative presentation in percentage enhances the understanding of both patients and their physicians regarding the meaning of liver fat percentage and the goal of reducing this measurement^1^. However, as previous studies have shown that B-mode US has low sensitivity in diagnosing hepatic steatosis, especially for mild steatosis^21^, further validation studies including people without steatosis or with mild hepatic steatosis, are required to establish their clinical performance in detecting and staging MASLD as a screening or diagnostic tool.

Therefore, this study aims to develop and validate a multivariable QUS parameters-derived estimated fat fraction for diagnosing hepatic steatosis in patients with MASLD, using prospectively collected QUS parameters from a tertiary hospital and a health screening center, with the MRI- PDFF as the reference standard.

## Methods

This was a retrospective cross-sectional study consisting of a secondary analysis of prospectively collected data from one tertiary hospitals and the other health screening center (IRB No. for the prospective data collection: 2002-020-1099 for Seoul National University Hospital [SNUH]; and 2022-05-054 for Samsung Medical Center [SMC]). This retrospective study was approved by the Institutional review board of those two hospitals (SNUH [IRB No. 2209-142-1362] and SMC [IRB No. 2023-01-022]), and performed in accordance with the Declaration of Helsinki. Written informed consent was waived for this retrospective analysis because of the nature of the study.

Two different study samples were used for developing and validating a statistical model to calculate US-based fat fraction from attenuation coefficient and backscatter coefficient, using MRI-PDFF as a reference standard.: a development study sample from SNUH (development set) in suspected MASLD population and a validation study sample from SMC (validation set) in health screening population.

### Study participants

#### Development set

**Figure 1:**
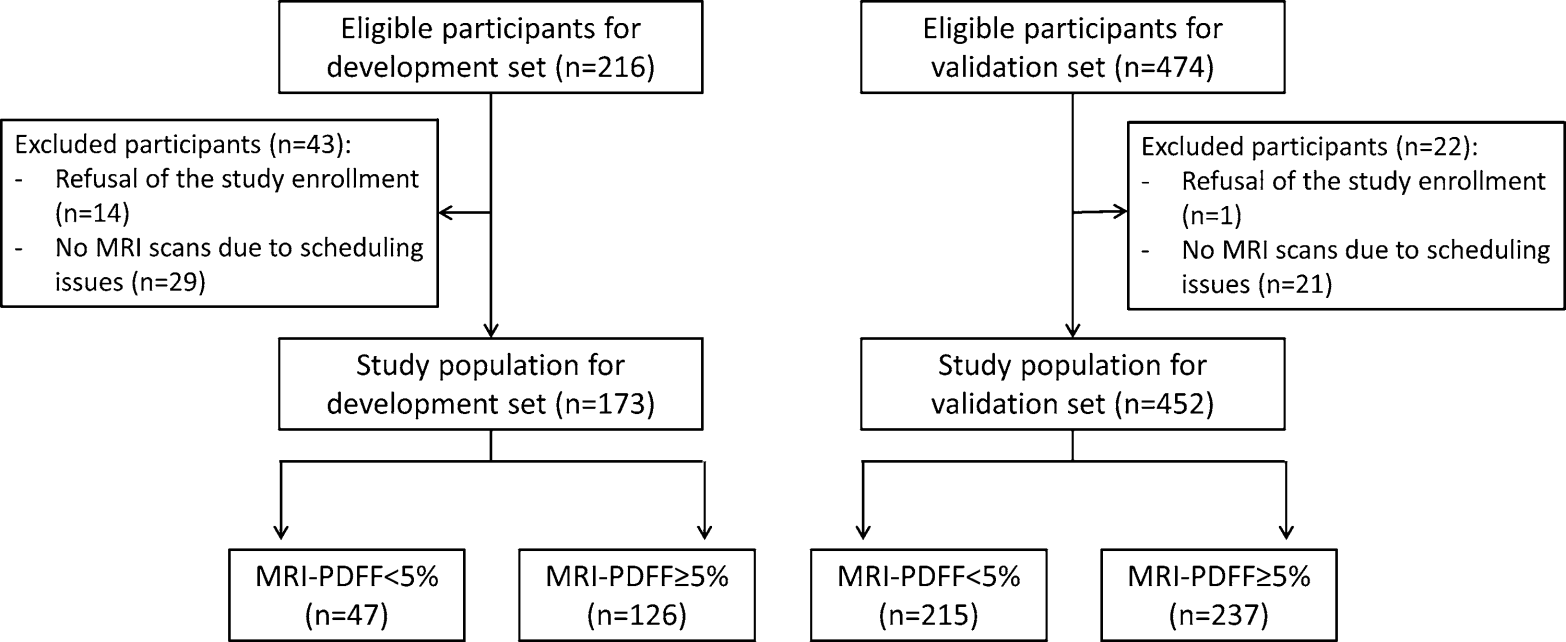
Flowchart of study population. *MRI*-*PDFF* magnetic resonance imaging proton density fat fraction.

Between July 2020 and June 2021, participants who were referred to the radiology department for liver US for known or clinically suspected MASLD or were scheduled to undergo hepatectomy for liver donation were prospectively enrolled in the study performed at SNUH. The enrolled participants were aged 18 years and above. The exclusion criteria of the study include (a) evidence of liver disease other than MASLD; (b) substantial alcohol consumption; (c) long-term use of hepatotoxic or steatogenic medication; (d) previous liver surgery; and (e) contraindication for MRI^18^ (Fig. 1).

#### Validation set

Between July 2022 and November 2022, participants who were 19 years or older and who underwent liver US for health screening were prospectively enrolled in the study performed at SMC. Among these participants, those who met the following exclusion criteria were excluded, which were: (a) evidence of chronic liver disease, including hepatitis B virus or hepatitis C virus-related diseases, (b) history of treatment for hepatic malignancy, (c) previous liver surgery, and (d) contraindication for MRI (Fig. 1).

### QUS examination

All US examinations were conducted by board-certified radiologists at each center (S.K.J., J.M.L., for SNUH and non-author radiologist for SMC with more than 9 years of experience, respectively). All participants underwent liver US examination using RS85 system (Samsung Medison, Seoul, Korea) using a convex probe (CA 1–7). All participants fasted at least 4 h before the US examination. After routine B-mode US examination of the liver parenchyma, QUS examination was performed in the right liver through the intercostal space. Participants were positioned in the supine position with the right arm at maximum abduction. For the measurement of two QUS parameters (tissue attenuation imaging [TAI] and tissue scatter-distribution imaging [TSI]), the operator placed a 2 × 3 cm fan-shaped region of interest (ROI) on the liver parenchyma, avoiding reverberation artifacts or large vessels. Areas with errors in parameter calculations, such as vascular structures, were automatically excluded from the calculation and presented as a vacancy on the TAI and TSI map. Operators obtained five measurements of QUS parameters (TAI and TSI) in each participant, which had a reliability index (R^2^ value) of 0.6 or larger according to the vendor’s recommendations. For each measurement, the five measurements were averaged as the representative value for each participant.

### Chemical shift-encoded MRI PDFF

All participants underwent chemical shift-encoded liver MRI by using one of two 3.0-T systems (MAGNETOM Skyra [Siemens Healthineers, Erlangen, Germany] for development set; and Ingenia CX [Philips Healthcare, Amsterdam, Netherlands] for validation set) on the day of US examination whenever possible, or within a 14-day period. Detailed MRI protocol and parameters are summarized in Supplementary Material 1. Blinded to QUS results, one person at each institution (S.K.J., for the development set; and non-author radiologist for the validation set) placed a 1-cm circular ROI on each of the nine Couinaud segments. MRI-PDFF values from liver segments 5–8 were averaged and used as the reference standard for hepatic fat content. Mild, moderate, and severe hepatic steatosis were defined using MRI thresholds of 5%, 15%, and 25%, respectively^22^.

### Statistical analysis

Demographic and imaging data of patients were compared between the development and validation sets using t-tests for continuous variables and Chi-square tests for categorical variables. Using the development data set, we developed a multivariable regression model using two QUS parameters, which provided an estimated fat fraction as percentage based on the generalized linear regression to predict PDFF. The correlation between QUS-derived estimated fat fraction (USFF) and MRI-PDFF was evaluated using the Pearson correlation coefficient. The agreement between USFF and MRI-PDFF was assessed using a Bland–Altman analysis with 95% limits of agreement (LOA). Additionally, to investigate factors associated with poor agreement between USFF and MRI-PDFF, we compared demographic and imaging data of patients between cases with good agreement (within 95% LOA) and cases with poor agreement (exceeding 95% LOA) using t-test (Supplementary Material 2). Receiver operating characteristic (ROC) curve analysis was used to evaluate the performance of USFF for detecting any hepatic steatosis (MRI-PDFF ≥ 5%), moderate to severe hepatic steatosis (MRI-PDFF ≥ 15%), and severe hepatic steatosis (MRI-PDFF ≥ 25%). Pairwise comparisons of the areas under the ROC curve (AUCs) between USFF and TAI or USFF and TSI were performed using Delong test. In this pairwise comparison analysis, a Bonferroni-adjusted P values of less than 0.017 (0.05/3) was considered to indicate statistical significance. Cutoff values were determined using the maximal Youden index^23^, and corresponding performance parameters, including sensitivity, specificity, positive predictive value (PPV), and negative predictive value (NPV) were calculated in the development set. Additionally, cutoff values of USFF for sensitivity and specificity exceeding 95% were also calculated in the development set. For the assessment of performance of all given cutoffs, the sensitivity, specificity, PPV, and NPV values were computed in the validation set. Additional analysis was conducted within a subgroup after performing propensity score matching for sex and age (n = 133 each for development and validation set each) for evaluating the performance of USFF in assessing hepatic steatosis (Supplementary Table 2). All statistical analyses were performed using MedCalc version 18.11.6 (MedCalc software, Ostend, Belgium). P < 0.05 was considered to indicate statistical significance.

## Results

### Participant characteristics

The development set included 173 participants (96 men and 77 women, mean age ± standard deviation, 51.1 years ± 14.1). The mean MRI-PDFF was 11.2% ± 7.8 and the mean body mass index was 26.5 kg/m^2^ ± 3.5. Out of the 173 participants, 126 (72.8%) were identified as having hepatic steatosis (MRI-PDFF ≥ 5%).

The validation set included 452 participants (380 men and 72 women, mean age ± standard deviation, 55.4 years ± 7.0). The mean MRI-PDFF was 7.9% ± 6.6, and the mean body mass index was 24.9 kg/m^2^ ± 2.9. Out of the 452 participants, 237 (52.4%) were identified as having hepatic steatosis (MRI-PDFF ≥ 5%). Participant characteristics of development and validation sets are summarized in Table 1.

**Table 1:** Participant characteristics.

Characteristics	Development set (n = 173)	Validation set (n = 452)	P value*
Age (years)	51.1 ± 14.1 (19–74)	55.4 ± 7.0 (35–78)	< 0.001
Sex			< 0.001
Male	96 (55.5)	380 (84.1)	
Female	77 (44.5)	72 (15.9)	
BMI (kg/m^2^)	26.5 ± 3.5 (19.2–39.8)	24.9 ± 2.9 (16.7–33.8)	< 0.001
Skin-to-liver capsule distance (mm)	20.7 ± 4.3 (11.0–35.0)	18.4 ± 3.3 (11.5–42.0)	< 0.001
Visual hepatic steatosis grade			< 0.001
S0	55 (31.8)	129 (28.5)	
S1	29 (16.8)	136 (30.1)	
S2	66 (38.2)	170 (37.6)	
S3	23 (13.3)	17 (3.8)	
MRI-PDFF (%)	11.2 ± 7.8 (1.5–46.4)	7.9 ± 6.6 (0.6–34.9)	< 0.001
< 5%	47 (27.2)	215 (47.6)	< 0.001
≥ 5 to < 15%	79 (45.7)	176 (38.9)	
≥ 15% to < 25%	37 (21.4)	47 (10.4)	
≥ 25%	10 (5.8)	14 (3.1)	

Values are presented as mean ± standard deviation (range) or number (%) as appropriate.

*NAFLD* nonalcoholic fatty liver disease, *BMI* body mass index, *MRI*-*PDFF* MRI proton density fat fraction.

*P values were calculated using a *t* test for continuous variables or Chi-square test for categorical variables.

### Univariable and multivariable regression model for fat fraction estimator

**Figure 2:**
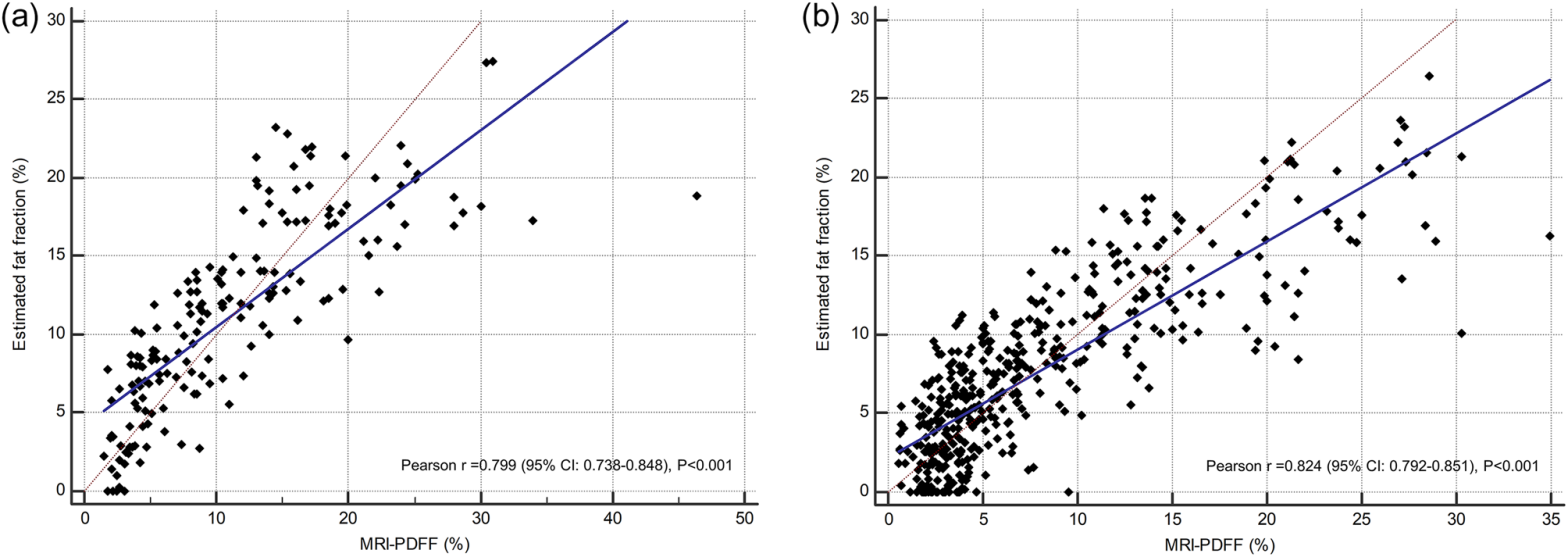
Multivariable QUS parameters-derived estimated fat fraction versus MRI-PDFF scatter plot, along with the identity line and linear regression line for development set (**a**) and validation set (**b**). *MRI*-*PDFF* magnetic resonance imaging proton density fat fraction, *CI* confidence interval.

Table 2 shows the results of univariable and multivariable regression analysis using TAI and TSI values for estimating hepatic steatosis with MRI-PDFF as reference standard. On univariable analysis, regression coefficients were 52.03 (95% CI 45.64, 58.42; P < 0.001) for TAI and 0.63 (95% CI 0.52, 0.75; P < 0.001) for TSI, and both two QUS parameters were selected for the multivariable model for fat fraction estimator. On multivariable regression analysis, USFF was calculated from the following equation: USFF = − 44.3 + 41.9*TAI + 0.23*TSI. If the value of USFF was negative, it was estimated as ‘zero’. USFF was correlated with MRI-PDFF in both development (r = 0.799 [95% CI 0.738–0.848], P < 0.001) and validation set (r = 0.824 [95% CI 0.792–0.851], P < 0.001). In the development set, linear regression of USFF against MRI-PDFF yielded a slope of 0.63 (95% CI 0.56–0.70), an intercept of 4.18 (95% CI 3.21–5.15), and R^2^ of 0.64 (Fig. 2). The Bland–Altman analysis showed a mean difference of 0% (95% limits of agreement [LOA], − 9.2 to 9.2%) between USFF and MRI-PDEFF for the development set, and − 0.3% (95% LOA, − 7.6 to 7.1%) for the validation set.

**Table 2:** Univariable and multivariable regression analysis for estimating hepatic fat fraction in development set.

Variable	Univariable analysis		Multivariable analysis*	
	Coefficient	P value	Coefficient	P value
TAI	52.03 (45.64, 58.42)	< 0.001	41.91 (33.93, 49.88)	< 0.001
TSI	0.63 (0.52, 0.75)	< 0.001	0.23 (0.12, 0.35)	0.001

Values in parentheses are 95% confidence intervals.

*TAI* tissue attenuation imaging, *TSI* tissue scatter-distribution imaging.

*Multivariable generalized linear regression model was developed using TAI and TSI, which provided an estimated fat fraction percentage, using magnetic resonance imaging proton density fat fraction as the reference standard.

### Diagnostic performance of QUS parameters and multivariable fat fraction estimator for hepatic steatosis

**Figure 3:**
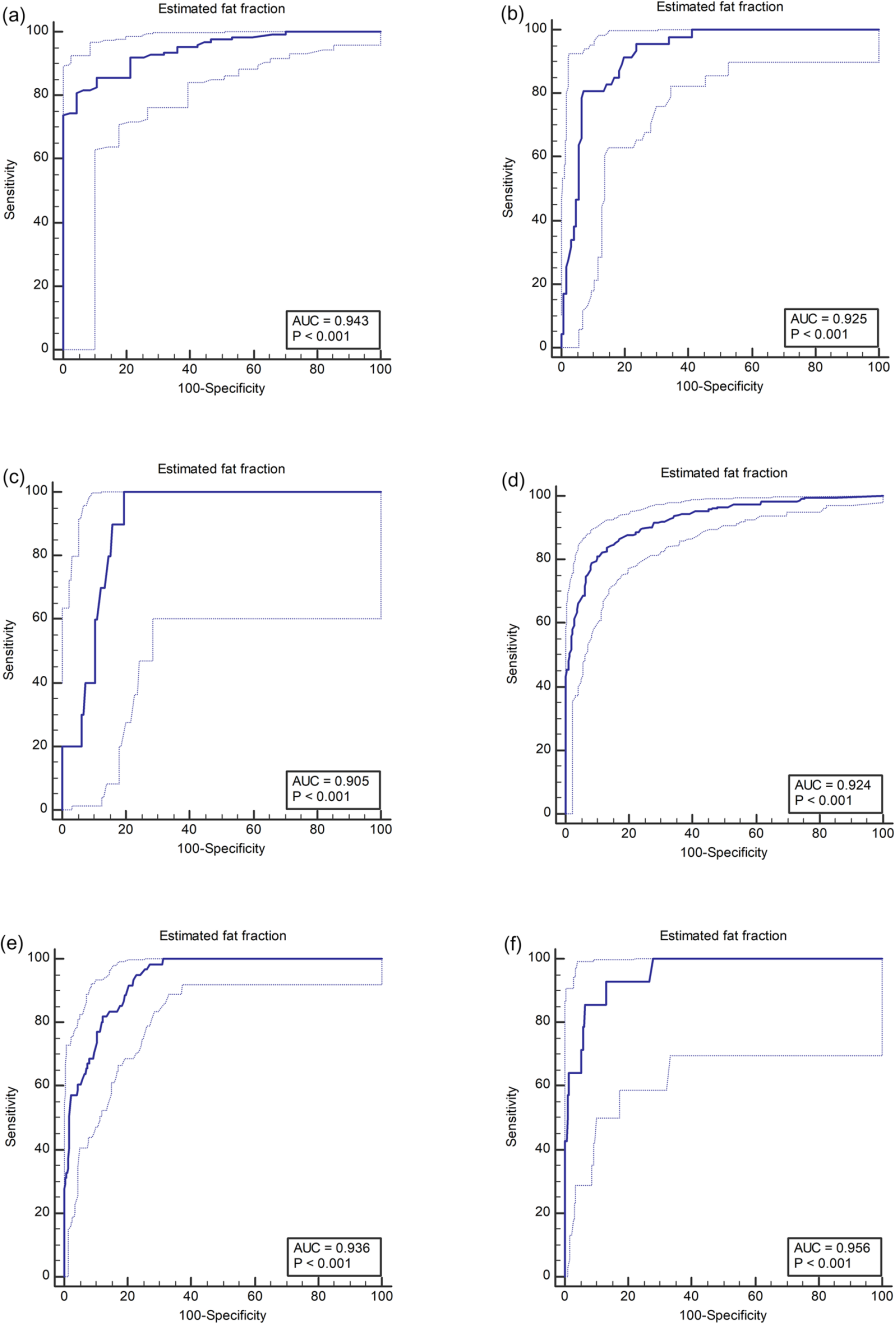
Diagnostic performance of multivariable QUS parameters-derived estimated fat fraction for diagnosing MRI-PDFF ≥ 5% (**a**,**b**),  ≥ 15% (**c**,**d**) and ≥ 25% (**e**,**f**) in development set (**a**,**c**,**e**) and validation set (**b**,**d**,**f**). *QUS* quantitative ultrasound, *MRI*-*PDFF* magnetic resonance imaging proton density fat fraction.

We assessed the diagnostic performance of TAI, TSI, and USFF derived from a multivariable regression model for assessing hepatic steatosis in the development and validation set (Table 3). Among three parameters, USFF showed the highest AUC for assessing MRI-PDFF ≥ 5%, ≥ 15%, and ≥ 25% in both development set (AUCs of 0.943, 0.925 and 0.905) and validation set (AUCs of 0.924, 0.936, and 0.956), respectively (Fig. 3).

**Table 3:** Diagnostic performance of QUS parameters and multivariable QUS-derived estimated fat fraction (USFF) for diagnosing hepatic steatosis.

	Development set	P value*	Validation set	P value*
	AUC (95% CI)		AUC (95% CI)	
MRI-PDFF ≥ 5%				
USFF (%)	0.943 (0.898, 0.973)		0.924 (0.895, 0.947)	
TAI (dB/cm/MHz)	0.917 (0.865, 0.953)	0.005	0.873 (0.838, 0.902)	< 0.001
TSI	0.905 (0.851, 0.944)	0.112	0.906 (0.876, 0.932)	0.141
MRI-PDFF ≥ 15%				
USFF (%)	0.925 (0.875, 0.959)		0.936 (0.909, 0.957)	
TAI (dB/cm/MHz)	0.914 (0.862, 0.951)	0.077	0.923 (0.895, 0.946)	0.012
TSI	0.843 (0.780, 0.894)	0.003	0.892 (0.859, 0.919)	0.007
MRI-PDFF ≥ 25%				
USFF (%)	0.905 (0.851, 0.944)		0.956 (0.933, 0.973)	
TAI (dB/cm/MHz)	0.898 (0.843, 0.939)	0.535	0.953 (0.929, 0.971)	0.614
TSI	0.813 (0.747, 0.868)	0.093	0.901 (0.870, 0.927)	0.033

*QUS* quantitative ultrasound, *AUC* area under the receiver operating characteristic curve, 95% *CI* 95% confidence interval, *MRI*-*PDFF* magnetic resonance imaging proton density fat fraction, *TAI* tissue attenuation imaging, *TSI* tissue scatter-distribution imaging.

*Receiver operating characteristic curve analysis was used to compute AUCs of USFF, TAI, and TSI in the evaluation of hepatic steatosis. A comparison of AUCs between USFF and TAI or USFF and TSI was conducted using Delong test, and a Bonferroni-adjusted P value of < 0.017 was considered to indicate statistical significance.

### Diagnostic performance of multivariable fat fraction estimator for hepatic steatosis in development and validation set

The performance of USFF for diagnosing hepatic steatosis in the development set and validation set are summarized in Table 4.

**Table 4:** Diagnostic performance of multivariable QUS-derived estimated fat fraction (USFF) for diagnosing hepatic steatosis in development and validation set.

	Cutoff (%)	Development set				Validation set			
		Sensitivity (%)	Specificity (%)	PPV (%)	NPV (%)	Sensitivity (%)	Specificity (%)	PPV (%)	NPV (%)
MRI-PDFF ≥ 5%									
Cutoff for maximal Youden index	8.7	81.0 (102/126, 73.0–87.4)	95.7 (45/47, 85.5–99.5)	98.1 (102/104, 92.9–99.5)	65.2 (45/69, 56.6–73.0)	68.8 (163/237, 62.5–74.6)	94.4 (203/215, 90.5–97.1)	93.1 (163/175, 88.6–96.0)	73.3 (203/277, 88.6–96.0)
Cutoff for 95% sensitivity (rule-out criteria)	6.0	95.2 (120/126, 89.9–98.2)	63.8 (30/47, 48.5–77.3)	87.6 (120/137, 82.8–91.2)	83.3 (30/36, 69.0–91.8)	87.8 (208/237, 82.9–91.7)	78.6 (169/215, 72.5–83.9)	81.9 (208/254, 77.7–85.4)	85.4 (169/198, 80.4–89.2)
Cutoff for 95% specificity (rule-in criteria)	8.7	81.0 (102/126, 73.0–87.4)	95.7 (45/47, 85.5–99.5)	98.1 (102/104, 92.9–99.5)	65.2 (45/69, 56.6–73.0)	68.8 (163/237, 62.5–74.6)	94.4 (203/215, 90.5–97.1)	93.1 (163/175, 88.6–96.0)	73.3 (203/277, 88.6–96.0)
MRI-PDFF ≥ 15%									
Cutoff for maximal Youden index	14.9	80.9 (38/47, 66.7–90.9)	92.1 (116/126, 85.9–96.1)	79.2 (38/48, 67.4–87.5)	92.8 (116/125, 87.8–95.9)	59.0 (36/61, 45.7–71.5)	95.7 (374/391, 93.1–97.4)	67.9 (36/53, 56.0–77.9)	93.7 (374/399, 91.7–95.3)
Cutoff for 95% sensitivity (rule-out criteria)	12.0	95.7 (45/47, 85.5–99.5)	76.2 (96/126, 67.8–83.3)	60.0 (45/75, 52.2–67.3)	98.0 (96/98, 92.5–99.5)	78.7 (48/61, 66.3–88.1)	88.2 (345/391, 84.6–91.3)	51.1 (48/94, 43.6–58.5)	96.4 (345/358, 94.2–97.7)
Cutoff for 95% specificity (rule-in criteria)	17.9	46.8 (22/47, 32.1–61.9)	95.2 (120/126, 89.9–98.2)	78.6 (22/28, 61.3–89.5)	82.8 (120/145, 78.5–86.3)	31.2 (19/61, 19.9–44.3)	99.2 (388/391, 97.8–99.8)	86.4 (19/22, 65.9–95.4)	90.2 (388/430, 8.6–91.6)
MRI-PDFF ≥ 25%									
Cutoff for maximal Youden index	16.0	100.0 (10/10, 69.2–100)	79.8 (130/163, 72.8–85.6)	23.3 (10/43, 18.3–29.1)	100.0 (130/130, 69.2–100)	78.6 (11/14, 49.2–95.3)	93.8 (411/438, 91.2–95.9)	29.0 (11/38, 20.5–39.1)	99.3 (411/414, 98.0–99.7)
Cutoff for 95% sensitivity (rule-out criteria)	16.0	100.0 (10/10, 69.2–100)	79.8 (130/163, 72.8–85.6)	23.3 (10/43, 18.3–29.1)	100.0 (130/130, 69.2–100)	78.6 (11/14, 49.2–95.3)	93.8 (411/438, 91.2–95.9)	29.0 (11/38, 20.5–39.1)	99.3 (411/414, 98.0–99.7)
Cutoff for 95% specificity (rule-in criteria)	23.2	20.0 (2/10, 2.5–55.6)	100 (163/163, 97.8–100)	100 (2/2, 97.8–100)	95.3 (163/171, 93.7–96.5)	14.3 (2/14, 1.8–42.8)	100 (438/438, 99.2–100)	100 (2/2, 99.2–100)	97.3 (438/450, 96.7–97.8)

Unless otherwise noted, data are percentages, with numerators and denominators and 95% confidence intervals in parentheses.

*PPV* positive predictive value; *NPV* negative predictive value; *MRI*-*PDFF* magnetic resonance imaging proton density fat fraction.

For diagnosing hepatic steatosis (MRI-PDFF ≥ 5%), the cutoffs of USFF were 6.0% for a sensitivity of greater than 95% and 8.7% for a specificity of greater than 95% in the development set. With the use of the dual cutoff approach, PPV and NPV in the development set were 87.6% (120/137, 95% CI 82.8–91.2) and 83.3% (30/36, 95% CI 69.0–91.8) with the use of cutoff for a sensitivity of greater than 95%. PPV and NPV in the development set were 98.1% (102/104, 95% CI 92.9–99.5) and 65.2% (45/69, 95% CI 56.6–73.0) with the use of cutoff for a specificity of greater than 95%. When these cutoffs were applied to the validation set, sensitivity, specificity, PPV, and NPV were 87.8% (208/237, 95% CI 82.9–91.7), 78.6% (169/215, 95% CI 72.5–83.9), 81.9% (208/254, 95% CI 77.7–85.4), and 85.4% (169/198, 95% CI 80.4–89.2) with the use of cutoff of 6.0%. Using a cutoff of 8.7%, sensitivity, specificity, PPV, and NPV were 68.8% (163/227, 95% CI 62.5–74.6), 94.4% (203/215, 95% CI 90.5–97.1), 93.1% (163/175, 95% CI 88.6–96.0), and 73.3% (203/277, 95% CI 88.6–96.0), respectively.

## Discussion

Our study demonstrated that estimated fat fraction from multivariable regression model using QUS parameters (USFF) was well correlated with MRI-PDFF (Pearson r = 0.799 and 0.824; for development and validation set, respectively), and the performance for diagnosing hepatic steatosis MRI-PDFF ≥ 5% was good in both development and validation set (AUCs of 0.943 and 0.924, for development and validation set, respectively). The good correlation between USFF and MRI-PDFF in a large number of study patients might have high clinical value and support clinical adoption of a less expensive modality with wider accessibility^24^. When applying a cutoff of 6.0%, which was derived from a development set (clinically suspected MASLD patients) for detection sensitivity of greater than 95% for MRI-PDFF ≥ 5% (rule-out criteria), USFF showed sensitivity, specificity, PPV, and NPV of 87.8%, 78.6%, 81.9%, and 85.4% for detecting hepatic steatosis in the validation set (health screening population). Although the previous study of US-derived fat fraction from other US vendors^17,24^ also showed similar diagnostic performance, the distinct significance of our study arise from its incorporation of screening population, including many patients without steatosis or with mild steatosis.. Based on our study results, as the USFF can provide good diagnostic accuracy for detecting and grading hepatic steatosis. We believe that the USFF technique can be used as a useful adjunct to B-mode ultrasound examination for screening hepatic steatosis.

Of interesting note, although the populations of our study (development and validation set) had different distributions in the prevalence of hepatic steatosis, the USFF showed similar diagnostic performance in both populations. Therefore, we believe that USFF can be used as a tool for diagnosing and screening hepatic steatosis. In addition, since our validation set included health screening population, it included both non-alcoholic and alcoholic fatty liver disease. Our results demonstrated that USFF can be a promising screening tool for patients with hepatic steatosis from various etiologies, not only MASLD. Furthermore, we calculated different cutoff levels (rule-out and rule-in criteria) for wide clinical applications. If used as a screening tool, a cutoff value that can improve sensitivity at the expense of specificity can be helpful. A cutoff value with optimal specificity can be helpful as a diagnostic tool. Using a dual cutoff level, USFF yielded PPVs of 98.1% and 93.1% for ruling in and NPVs of 83.3% and 85.4% for ruling out patients with hepatic steatosis in the development and validation sets, respectively.

In addition, the USFF is expressed as a percentage, analogous to MRI-PDFF^17,24^. This presentation of USFF is helpful in improving communication with patients and clinicians and might be beneficial for longitudinal therapy monitoring. Previous studies of convolutional neural network using QUS maps and ultrasound radiofrequency data also demonstrated excellent study results in assessing hepatic steatosis^18,20^. However, their reliance on extensive computing resources and databases poses significant challenges for practical implementation in clinical settings. Therefore, considering the ease of development and clinical application, statistical methods such as multivariable regression model can be preferred in such contexts. In line with this, efforts are being made to develop statistical models that propose US-based fat fraction in clinical vendors. Compared with those studies, which required deep learning models and RF US images, our study used multiple regression model using TAI and TSI measurement results and resulted in more straightforward and rapid calculations. Considering US as the most used imaging test for evaluating diffuse liver diseases and is also inexpensive, easily accessible imaging test with QUS measurements available in few seconds, the USFF seems to be promising for being used as a tool for screening hepatic steatosis.

In terms of grading of hepatic steatosis, among TAI, TSI, and USFF, USFF showed the highest AUC for assessing MRI-PDFF ≥ 5%, ≥ 15%, and ≥ 25% in both development and validation sets. We found that the diagnostic performance of all three parameters in assessing MRI-PDFF ≥ 25% was worse than MRI-PDFF ≥ 5% or ≥ 15%. These results are similar to previous studies^18,25^, and can be explained by the limitation of US signals which are weak in severe hepatic steatosis since it is already attenuated in the nearfield^20^. Therefore, we believe that QUS-derived estimated fat fraction is suitable as screening test or diagnostic test for mild or moderate degree steatosis.

Our study has several limitations. First, although both the development and validation set were originally obtained from prospective data, the patient characteristics of both groups were significantly different. However, the validation group included a large number of patients without hepatic steatosis; this might be even more valuable. Second, although the sample size was large in both groups, all patients were from the same ethnic group, and therefore, the study is limited in terms of demographic and clinical variables. Third, additional studies are needed to validate the diagnostic performance of USFF against liver biopsy. Fourth, in this study, we evaluated a single vendor’s ultrasound system. Further research using ultrasound system from various vendors is needed to validate our study results. Fifth, our study could not evaluate the liver stiffness value (MR elastography or shear-wave elastography data) of patients. To achieve a more comprehensive understanding of the disease status in steatotic liver disease, it would be valuable to conduct additional validation studies that encompass both USFF and liver stiffness measurements. Finally, as our study did not include repeated measurements of USFF with an interval, we could not evaluate the capability of USFF for monitoring therapeutic effect of lifestyle changes or drug interventions.

In conclusion, multivariable QUS parameter-derived estimated fat fraction showed a good correlation with MRI-PDFF and high diagnostic performance for detecting hepatic steatosis in both development set of patients with suspected MASLD and validation data set of health screening population. Therefore, it can potentially be used as a screening imaging test to evaluate hepatic steatosis.

### Supplementary Information

Supplementary Information.

## Data Availability

The data used to support the findings of this study are available from the corresponding author on reasonable request.
