# Supplementary material for: Development and validation of multivariable quantitative ultrasound for diagnosing hepatic steatosis
Source: Sci Rep. 2023 Sep 14;13:15235. doi: 10.1038/s41598-023-42463-w (PMC10502048; doi:10.1038/s41598-023-42463-w)
Supplement: Supplementary file 1 — Supplementary Information. [file 41598_2023_42463_MOESM1_ESM.docx]

**Development and validation of multivariable quantitative ultrasound for diagnosing hepatic steatosis**

Manuscript type: Original Research

Sun Kyung Jeon^1^, Jeong Min Lee^1,2*^, Soo Jin Cho^3*^, Young-Hye Byun^3^, Jae Hwan Jee^3^, Mira Kang^3,4,5^

Department of Radiology, Seoul National University Hospital and Seoul National University College of Medicine^1^

Institute of Radiation Medicine, Seoul National University Medical Research Center, Seoul, Korea^2^

Center for Health Promotion, Samsung Medical Center, Sungkyunkwan University School of Medicine, Seoul, Korea^3^

Department of Digital Health, Samsung Advanced Institute of Health Sciences & Technology (SAIHST), Sungkyunkwan University, Seoul, Korea^4^

Digital Innovation Center, Samsung Medical Center, Sungkyunkwan University School of Medicine, Seoul, Korea^5^

*Co-corresponding authors

Corresponding authors:

**Jeong Min Lee, MD, PhD**

Department of Radiology, Seoul National University Hospital and Seoul National University College of Medicine

101 Daehangno, Jongno-gu, Seoul, 03080, Korea

Tel: 82-2-2072-2057; Fax: 82-2-743-6385

E-mail: Jeong Min Lee, [jmsh@snu.ac.kr](mailto:jmsh@snu.ac.kr)

**Soo Jin Cho, MD**

Center for Health Promotion, Samsung Medical Center, Sungkyunkwan University School of Medicine

81 Irwon-ro, Gangnam-Gu, Seoul, 06351, Korea

Tel: 82-2-3410-3885 ; Fax: 82-2-3410-0054

E-mail: Soo Jin Cho, [soojin77.cho@samsung.com](mailto:soojin77.cho@samsung.com)

**Supplementary Material 1. Details of MRI protocol and parameters**

All participants underwent chemical shift-encoded liver MRI by using one of two 3.0-T systems (MAGNETOM Skyra [Siemens Healthineers, Erlangen, Germany] for development set; and Ingenia CX [Philips Healthcare, Amsterdam, Netherlands] for validation set) on the day of US examination whenever possible, or within a 14-day period. Six two-dimensional gradient-echo images were obtained using complex-based chemical shift-encoded water-fat reconstruction techniques, and PDFF maps were reconstructed automatically using a vendor-specific algorithm with T2* correction calculated from single decay and multipeak fat model [1]. Detailed MRI parameters of the chemical shift-encoded MRI-PDFF of each institution are summarized in Supplementary Table 1.

**Supplementary Material 2. Factors associated with poor agreement between USFF and MRI-PDFF**

Patients demographics and imaging data were compared between cases with good agreement between USFF and MRI-PDFF (within Bland-Altman 95 % limits of agreement; n=595) and cases with poor agreement between USFF and MRI-PDFF (exceeding Bland-Altman 95% limits of agreement; n=30). Patients with poor agreement between USFF and MRI-PDFF showed higher MRI-PDFF values (23.1 ± 9.4% vs. 8.1 ± 6.2%, P<0.001), higher USFF values (13.3 ± 4.4% vs. 8.4 ± 5.9%, P<0.001), higher BMI (26.6 ± 2.7kg/m^2^ vs. 25.3 ± 3.2kg/m^2^, P=0.031), and larger skin-to-liver capsular distance (2.1 ± 0.4 cm vs. 1.9 ± 0.4 cm, P=0.003) than patients with good agreement between USFF and MRI-PDFF.

**REFERENCES**

1. Hamilton G, Yokoo T, Bydder M et al. In vivo characterization of the liver fat ¹H MR spectrum. NMR in biomedicine 2011; 24: 784-790. DOI: 10.1002/nbm.1622

**Supplementary Table 1. Details of MRI parameters**

| MR machine | TR (msec) | TE (msec) | Matrix | Slice thickness (mm) | Flip angle (°) |
| --- | --- | --- | --- | --- | --- |
| Skyra (Siemens Healthineers) | 9.3 | 1.3 | 256x192 | 3 | 4 |
| Ingenia CX (Philips Healthcare) | 6.1 | 1.06 | 160x160 | 4 | 3 |

Note. QUS =- quantitative ultrasound, AUC = area under the receiver operating characteristic curve. 95% CI = 95% confidence interval. MRI-PDFF = magnetic resonance imaging proton density fat fraction. TAI = tissue attenuation imaging, TSI = tissue scatter-distribution imaging.

**Supplementary Table 2. Subgroup analysis of diagnostic performance of multivariable QUS-derived estimated fat fraction (USFF) for diagnosing hepatic steatosis (propensity score matching, N=133 for development and validation set each)**

|  | Development set (n=133)^*^ | | | | | | Validation set (n=133)^*^ | | | | |
| --- | --- | --- | --- | --- | --- | --- | --- | --- | --- | --- | --- |
|  | AUC  (95% CI) | Cutoff (%) | Sensitivity (%) | Specificity (%) | PPV (%) | NPV (%) | AUC  (95% CI) | Sensitivity (%) | Specificity (%) | PPV (%) | NPV (%) |
| MRI-PDFF ≥ 5% | 0.939 (0.884, 0.973) | 8.7 | 84.0 (84/100) | 93.9  (31/33) | 97.7  (84/86) | 66.0  (31/47) | 0.945 (0.892, 0.977) | 72.1  (44/61) | 95.8  (69/72) | 93.6 (44/47) | 80.2 (69/86) |
| MRI-PDFF ≥ 15% | 0.909 (0.847, 0.952) | 14.9 | 75.0  (27/36) | 91.8  (89/97) | 77.1 (27/35) | 90.8  (89/98) | 0.950 (0.898, 0.980) | 60.0 (9/15) | 95.8  (113/118) | 64.3  (9/14) | 95.0  (113/119) |
| MRI-PDFF ≥ 25% | 0.921 (0.861, 0.960) | 16.0 | 100  (10/10) | 82.1  (101/123) | 31.3  (10/32) | 100  (101/101) | 0.967 (0.921, 0.990) | 75.0  (3/4) | 94.6  (122/129) | 30.0  (3/10) | 99.2  (122/123) |

Note. QUS = quantitative ultrasound, AUC = area under the receiver operating characteristic curve, 95% CI = 95% confidence interval, PPV = positive predictive value, NPV = negative predictive value, MRI-PDFF = magnetic resonance imaging proton density fat fraction. Unless otherwise noted, data are percentages, with numerators and denominators in parentheses.^*^133 patients consisted 70 men and 64 women for both development and validation set, with no significant difference in age (56.4±9.1 years vs. 56.3±8.7 years, P=0.896).
